# HGF–Met Pathway in Regeneration and Drug Discovery

**DOI:** 10.3390/biomedicines2040275

**Published:** 2014-10-31

**Authors:** Kunio Matsumoto, Hiroshi Funakoshi, Hisaaki Takahashi, Katsuya Sakai

**Affiliations:** 1 Division of Tumor Dynamics and Regulation, Cancer Research Institute, Kanazawa University, Kanazawa 920-1192, Japan; E-Mail: k_sakai@staff.kanazawa-u.ac.jp; 2 Center for Advanced Research and Education, Asahikawa Medical University, Asahikawa 078-8510, Japan; E-Mails: hfuna@asahikawa-med.ac.jp (H.F.); hisaaki@asahikawa-med.ac.jp (H.T.)

**Keywords:** amyotrophic lateral sclerosis, clinical trial, HGF, Met, spinal cord injury

## Abstract

Hepatocyte growth factor (HGF) is composed of an α-chain and a β-chain, and these chains contain four kringle domains and a serine protease-like structure, respectively. Activation of the HGF–Met pathway evokes dynamic biological responses that support morphogenesis (e.g., epithelial tubulogenesis), regeneration, and the survival of cells and tissues. Characterizations of conditional *Met* knockout mice have indicated that the HGF–Met pathway plays important roles in regeneration, protection, and homeostasis in various cells and tissues, which includes hepatocytes, renal tubular cells, and neurons. Preclinical studies designed to address the therapeutic significance of HGF have been performed on injury/disease models, including acute tissue injury, chronic fibrosis, and cardiovascular and neurodegenerative diseases. The promotion of cell growth, survival, migration, and morphogenesis that is associated with extracellular matrix proteolysis are the biological activities that underlie the therapeutic actions of HGF. Recombinant HGF protein and the expression vectors for HGF are biological drug candidates for the treatment of patients with diseases and injuries that are associated with impaired tissue function. The intravenous/systemic administration of recombinant HGF protein has been well tolerated in phase I/II clinical trials. The phase-I and phase-I/II clinical trials of the intrathecal administration of HGF protein for the treatment of patients with amyotrophic lateral sclerosis and spinal cord injury, respectively, are ongoing.

## 1. Background of Hepatocyte Growth Factor (HGF)–Met Pathway Leading to Drug Discovery

HGF was molecularly cloned as a growth factor for hepatocytes [1,2]. The scatter factor, originally identified as a fibroblast-derived cell motility factor for epithelial cells [3], was shown to be an identical molecule to HGF [4]. The receptor for HGF was identified as a c-met protooncogene product of transmembrane receptor tyrosine kinase in 1991 [5,6]. These early findings implicated biological and pathophysiological roles for HGF in epithelial wound healing, tissue regeneration, tumorigenesis, and cancer invasion.

**Figure 1:**
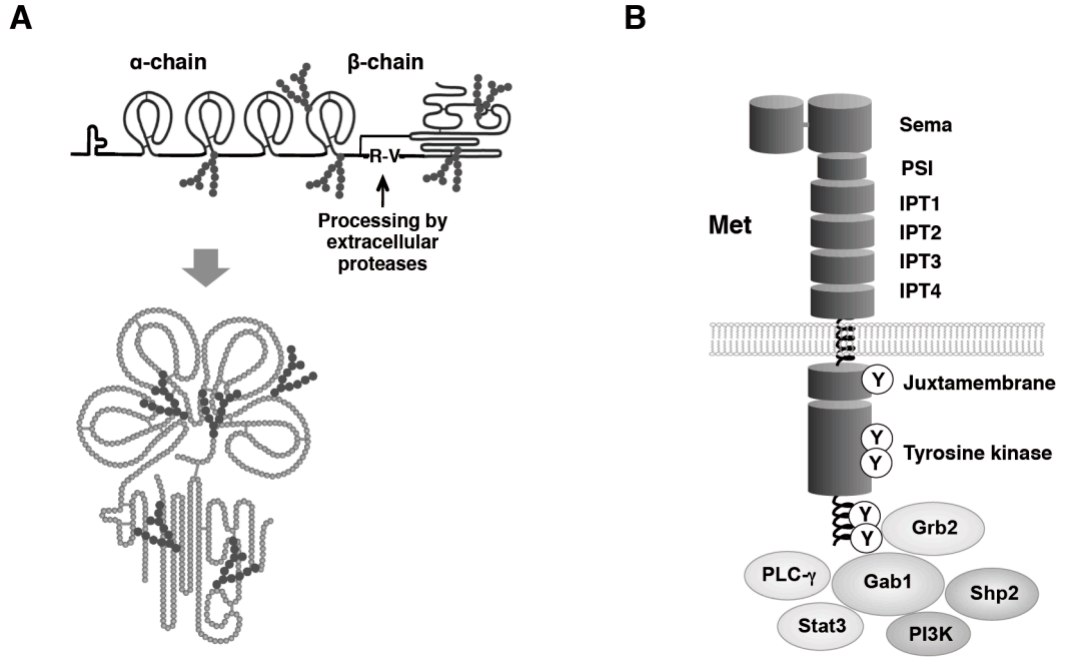
Schematic structures of HGF (**A**) and Met (**B**). A single-chain HGF is cleaved between Arg494 and Val495 by serine proteases and HGF is modified by glycosylation. Domain structure of Met and typical signaling molecules are described.

HGF is a glycosylated protein composed of an α-chain and a β-chain linked by one disulfide bridge (Figure 1A) [1,2]. HGF is biosynthesized as a prepro-form, including a signal sequence and both α- and β-chains. After cleavage of a signal peptide of the first 31 amino acids and extracellular secretion, a single-chain HGF is further cleaved between Arg494 and Val495 by serine proteases such as HGF-activator, matriptase, and hepsin [7,8]. Mature HGF is composed of 697/692 amino acids.

The Met receptor is composed of structural domains that include the extracellular Sema (the domain found in semaphorin receptors), the PSI (the domain found in plexins, semaphorins and integrins), the IPT (the domain found in immunoglobulins, plexins, and transcription factors), the transmembrane, the intracellular juxtamembrane, and the tyrosine kinase domains (Figure 1B) [9]. The Sema domain serves as a key element for ligand binding [10], while the involvement of IPT-3 and IPT-4 in the binding to HGF was demonstrated by another approach [11].

Binding of HGF to the Met results in the phosphorylation of multiple tyrosine residues within the cytoplasmic region. The phosphorylation of Tyr1234 and Tyr1235 within the tyrosine kinase domain positively regulates the catalytic activity of tyrosine kinase, and the subsequent phosphorylation of *C*-terminal tyrosine residues Tyr1349 and Tyr1356 recruits intracellular signaling molecules, including growth factor receptor-bound protein 2 (Grb2), GRB2-associated-binding protein 1 (Gab1), phosphoinositide 3-kinase (PI3K), SH2 containing protein tyrosine phosphatase (Shp2), phospholipase Cγ1 (PLCγ1), and signal transducer and activator of transcription-3 (Stat3). Typical biological activities evoked by Met activation include promotion of mitogenesis and migration, suppression of cell death, and induction of epithelial morphogenesis. Among signaling molecules, Gab1 plays a critical role in HGF–Met pathway-dependent biological responses [12,13]. Gab1 is a scaffolding adaptor containing the Met-binding site, by which a direct and robust interaction between Gab1 and Met results in prolonged Gab1 phosphorylation in response to HGF. Association of Gab1 to Met is responsible for unique biological activities of HGF, including epithelial tubulogenesis. Gab1^−/−^ and Met^−/−^ mice were characterized by impairment in migration of myogenic precursor cells and formation of functional placenta.

**Figure 2:**
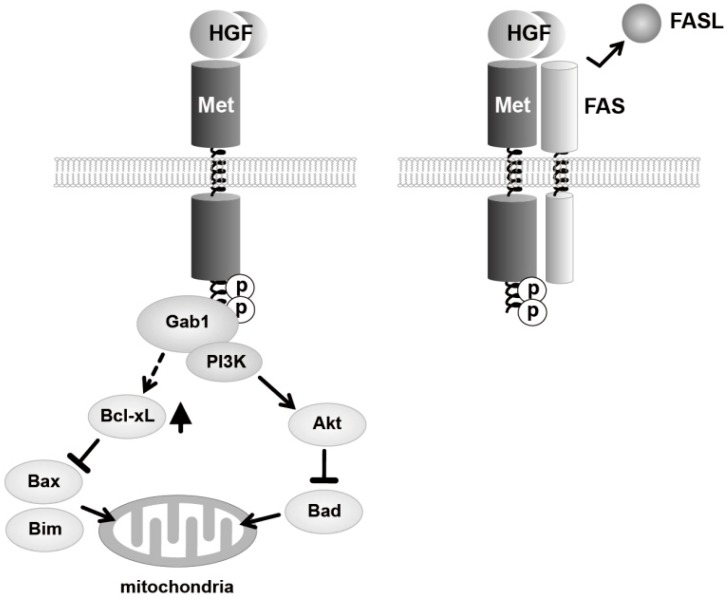
Signaling mechanisms responsible for promotion of cell survival mediated by HGF–Met pathway.

Promotion of cell survival (*i.e.*, suppression of cell death) is a key biological action of HGF–Met pathway in development, regeneration, and therapeutics. Activation of Met induces phosphorylation of PI3K and Akt, and Akt-induced phosphorylation of Bad inhibits pro-apoptotic activity of Bad (Figure 2). In parallel, HGF increases expression anti-apoptotic Bcl-xL, by which pro-apoptotic activity of Bax and Bim is inhibited [14,15]. Fas and Fas-ligand play a marked role in induction of apoptosis. Another anti-apoptotic mechanism involves extracellular interaction between the extracellular domain of Met and the death receptor FAS [16]. Binding of HGF to Met prevents functional association between FAS and FAS-ligand, therefore inhibiting the self-aggregation of FAS, a key event required for induction of FAS-mediated apoptosis.

The cytoplasmic juxtamembrane domain negatively regulates Met-dependent signal transduction. Cbl ubiquitin ligase binds phosphorylated Y1003, and this Cbl binding results in Met ubiquitination and degradation [17]. Phosphorylation of Ser985 in the juxtamembrane domain also regulates Met activation. Ser985 is phosphorylated by protein kinase-C, and when Ser985 is phosphorylated, HGF-dependent Met tyrosine phosphorylation is suppressed [18].

**Figure 3:**
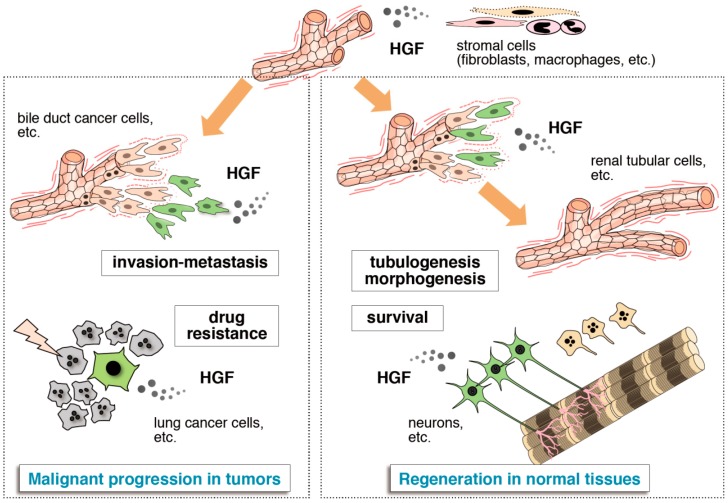
Two-pronged roles of HGF in tissue regeneration and cancer tissues. HGF is mainly expressed in stromal cells. Cells responding to HGF are conceptually shown in green. Dynamic morphogenesis (e.g., blanching tubulogenesis in renal tubular cells) and promotion of cell survival (e.g., for neurons) mediated by the HGF–Met pathway play roles in tissue regeneration after tissue injury (**right part**). Dynamic cell movement and survival promoted by Met activation participate in invasion-metastasis and resistance to anticancer drugs in cancer tissues (**left part**).

In most cases in the relationship between growth factors and their receptors, a single growth factor activates multiple receptors that have structural similarities, while a single receptor has multiple ligands with structural similarities. By contrast, the sole receptor for HGF is Met, while the sole ligand for Met is HGF; the relationship is a “one-to-one relationship”. This unique biochemical characteristic in the HGF–Met pathway promotes drug development by targeting HGF–Met through either the activation or the inhibition of the HGF–Met pathway. HGF has biological activities involved in dynamic tissue remodeling during embryogenesis and tissue regeneration. Breakdown of the extracellular matrix scaffold and the concomitant cellular migration, mitogenesis, and morphogenesis that is driven by the HGF–Met pathway all make way for the construction and reconstruction of tissues. HGF suppresses cell death and promotes the survival of cells, and this action participates in the protection of cells and tissues against injuries and pathology. However, these biological actions that are driven by the HGF–Met pathway all play a role in the acquisition of the malignant characteristics in tumor cells—invasion, metastasis, and drug resistance in the tumor microenvironment (Figure 3).

## 2. Tissue Regeneration/Protection Deduced from Met Disruption

In conventional knockout mice of the *HGF* or *Met* gene in whole body is lethal during the embryonic stage due to an impaired organogenesis of the placenta and liver [19,20]. Moreover, HGF provides spatially defined chemoattractant-like motogenic signals for myogenic precursor cells. The migration of myogenic precursor cells from the dermo-myotome in the somite to the limb buds and diaphragm is impaired in Met^−/−^ mice. With this condition, the skeletal muscles of the limbs and diaphragm are not formed in mutant mice [21].

Definitive roles of the HGF–Met pathway in tissue protection and repair have been demonstrated using a conditional knockout of the *Met* gene in mice (Table 1). Hepatocytes subjected to selective loss of the functional Met were highly susceptible to cell death even after mild liver injury, indicating that the anti-apoptotic activity of HGF plays a role in the protection of the liver [22]. Liver-specific Met^−/−^ mice showed delayed liver regeneration associated with persistent inflammatory reaction [23]. Activation of Met plays a role in the persistent Erk1/2 activation and the G2/M gene expression program throughout liver regeneration following partial hepatectomy [24]. In addition to the regenerative response in mature hepatocytes, HGF–Met signaling supports the *in vitro* sphere formation of hepatic stem cells (oval cells) and *in vivo* hepatic stem cell-mediated regeneration [24]. Met-deficient oval cells were more prone to apoptosis when the cells were exposed to proapoptotic conditions after bile duct ligation. The livers in hepatocyte-specific Met^−/−^ mice were more susceptible to chronic inflammation and fibrotic change compared with control mice [25]. The effects shown by these liver- or hepatocyte-specific Met^−/−^ mice indicate the physiological roles of the HGF–Met pathway in the protection, regeneration, anti-inflammation, and anti-fibrosis of the liver.

**Table 1:** Physiological roles of HGF deduced from conditional knockout mice.

Met^−/−^ Tissue/Cell Types	Characteristics	Ref.
**Liver**		
Hepatocytes	Highly susceptible to apoptosis after liver injury	[22]
	Impairment in recovery from liver necrosis after liver injury	
	Impairment in Erk1/2 activation and G2/M transition after liver injury	[23]
Hepatocytes	Steatotic change of the liver in aged mice	[24]
	Decrease in mitotic hepatocytes after partial hepatectomy	
	Delayed regeneration after partial hepatectomy	
Hepatocytes	Promoted liver fibrosis after liver injury	[26]
	Extensive necrosis and lower proliferation of hepatocytes after bile-duct ligation	[25]
	Enhanced susceptibility to liver fibrosis	
Oval cells	Decrease in oval cell viability and more prone to apoptosis	[27]
	Reduction in oval cell pool	[28]
	Impairment in migration and differentiation into hepatocytes	
**Kidney**		
Tubular cells	No appreciable defect in kidney morphology and function	[29]
	Aggravated renal injury and inflammation after acute kidney injury	
Podocytes	Neither albuminuria nor overt pathologic lesions	[30]
	Severe podocyte injury and apoptosis, and albuminuria after toxic injury	
Collecting duct	Increased fibrosis and tubular necrosis after unilateral ureteral obstruction	[31]
	Reduced capacity in regeneration after release of the obstruction	
Ureteric bud	Double knockout of *Met* and *EGF* receptor in ureteric bud	[32]
	Decrease in branching and a reduction in final glomerular number	
**Skin**		
Keratinocytes	Lack of keratinocyte migration after skin wound	[33]
	Severe impairment epidermal wound closure	
**Pancreas**		
β-Cell	Mild hyperglycemia, and decreased serum insulin levels at 6 months	[34]
	Loss of acute-phase insulin secretion in response to glucose, and impaired glucose tolerance	
	Diminished glucose tolerance and reduced plasma insulin after a glucose challenge	[35]
	Normal glucose and β-cell homeostasis	[36]
	Susceptible to streptozotocin-induced diabetes	
**Nervous System**		
Ganglionic eminence	Increased numbers of striatal GABAergic interneurons in the lateral sensorimotor	[37]
	Areas with distinct behavioral deficits	
	Delayed procedural learning	
Cerebral cortex and hippocampus	Larger size in the rostral cortex, caudal hippocampus, dorsal striatum, thalamus, and corpus callosum	[38]
Dorsal pallial	Increases proximal and reduces distal apical dendritic branching of neocortical pyramidal neurons in post-pubertal period	[39]
Forebrain neurons	Reduced volume of cortical tissue	[40]
	Increase in spine head volume, but no change in density of spines	
	Hyperconnectivity in circuit-specific intracortical neurons	
**Heart**		
Cardiomyocytes	Normal heart development	[41]
	Cardiomyocyte hypertrophy and interstitial fibrosis by 6 months	
	Systolic cardiac dysfunction by 9 months	
**Immune System**		
Dendritic cells	Impaired emigration toward draining lymph nodes upon inflammation-induced activation	[42]
	Impaired contact hypersensitivity reaction to contact allergens	

Characterization of conditional *Met* knockout mice indicates that the HGF–Met pathway plays important roles in regeneration, protection, and homeostasis in various cells and tissues (Table 1). The loss of functional Met in renal tubules caused no appreciable defect in renal function. However, when mice were subjected to renal injury, tubular cell-specific Met^−/−^ mice displayed higher serum creatinine, greater severity in morphologic lesions, and an increase in apoptosis compared with control mice [29]. In podocyte-specific Met^−/−^ mice, no pathology was seen, but when subjected to toxic renal injury of the podocytes, these mice developed podocyte apoptosis and albuminurea that was more severe compared with that of control mice [30]. Collective duct-selective Met dysfunction indicated a trend toward increased interstitial fibrosis, infiltration of the interstitium, and acute tubular necrosis after unilateral obstruction, while there was a reduced regenerative response after the release of obstruction [31].

Disruption of the *Met* gene in epidermal keratinocytes demonstrated an indispensable role for the HGF–Met pathway in skin wound healing [28]. Because the migration of keratinocytes post-wounding was almost completely impaired in Met^−/−^ keratinocytes, re-epithelialization was severely suppressed. Wound closure occurred exclusively in a few keratinocytes that had escaped recombination, which indicated that the skin wounding process had selected and amplified residual cells that expressed a functional Met. Those results indicated a definitive role for the HGF–Met pathway in skin wound healing. In mice with Met-deficient dendritic cells, Met-deficient dendritic cells failed to reach skin-draining lymph nodes upon activation while exhibiting an activated phenotype, and the contact hypersensitivity reactions in response to contact allergens was greatly impaired [42]. HGF–Met signaling in cutaneous dendritic cells may play a critical role in the maintenance of normal immune function.

Conditional knockout mice with selective disruption of *Met* in pancreatic β-cells displayed significantly diminished glucose tolerance and reduced plasma insulin after a glucose challenge [35]. *In vitro* glucose-stimulated insulin secretion in the islets from β-cell-Met^−/−^ mice was decreased by ~50% compared with control islets. These changes in β-cell function in the conditional *Met* knockout mice were not accompanied by changes in total β-cell mass, islet morphology, or β-cell proliferation [35]. Another group using β-cell-Met^−/−^ mice displayed mild hyperglycemia and a complete loss of acute-phase insulin secretion in response to glucose [34]. Therefore, HGF–Met signaling in the β-cell is not essential for β-cell growth, but it is essential for normal glucose-dependent insulin secretion and glucose homeostasis.

## 3. Neurotrophic Function and Involvement in Neuronal Disorder/Symptoms

HGF also functions as a novel neurotrophic factor for a variety of neurons, including the hippocampal, cerebral cortical, midbrain dopaminergic, motor, sensory, sympathetic, parasympathetic, and cerebellar granule neurons in culture [43]. Knock-out/-in studies and stereotaxic injections of anti-HGF antibody into the striatum have revealed the critical role of HGF in the nervous system [43]. HGF plays important roles in motor (including muscles), sensory, sympathetic, parasympathetic, and cortical neuronal development. In addition to the neuronal development, intrastriatal injections of anti-HGF IgG has revealed the involvement of HGF in the proliferation of oligodendrocyte progenitor cells and their differentiation into oligodendrocytes [44].

Approaches to address the susceptibility genes in neuronal disorders have revealed a relationship between Met transcriptional regulation and autism. The genetic association of a common C allele in the promoter region of the *Met* gene was found in 204 families with autism [45]. The allelic association for this Met variant was confirmed in a replication sample of 539 families with autism and in a combined sample. These researchers also found that Met protein levels were significantly decreased in autism spectrum disorder cases, compared with control subjects. Their subsequent study supported the association of the Met promoter variant rs1858830 C allele with autism spectrum disorder, implying a promoter mutation in autism spectrum disorder [46]. New mutations were noted in autism, in both single locus and haplotype approaches, with a single nucleotide polymorphism in intron 1 and with one intronic haplotype, in 325 multiplex International Molecular Genetics of Autism Consortium families [47].

The molecular mechanism by which mutations found in the *Met* gene become causative for characteristics seen in autism and autism spectrum disorder should be further addressed, but the neuro-developmental role of the HGF–Met pathway may be somewhat altered by these mutations in the *Met* gene. The HGF–Met pathway contributes to the development of the cerebral cortex [48,49] and the cerebellum [50], both of which exhibit developmental disruptions in autism [51,52]. Hypomorphic Met–HGF signaling in the cerebral cortex results in abnormal interneuron migration from the ganglionic eminence and reduced interneurons in the frontal and parietal regions of the cortex [48,49]. Hypomorphic HGF–Met signaling in the cerebellum causes a decrease in the proliferation of granule cells and a concomitant reduction in the size of the cerebellum, particularly in the vermis [50]. Both of these neuropathologic abnormalities are consistent with those observed in the brains of individuals with autism [51,52]. These results show why the *Met* gene is pursued as an autism candidate gene.

A comparison of 21 single nucleotide polymorphisms in the *Met* gene of 173 Caucasian patients with schizophrenia status and 137 controls revealed an association between genetic variation in the *Met* gene and schizophrenia [53]. Genetic variations of the Met are associated with general cognitive ability. These findings implicated the HGF–Met pathway in psychiatric status and diseases.

Three mutations in the *HGF* gene were found in patients with nonsyndromic hearing loss, at the autosomal-recessive NSHI locus *DFNB39* [54]. Two of the mutations occurred in a region contained in the 3'UTR of an alternate splice form of the *HGF* gene that had not been previously discovered. The third mutation, a third-position nucleotide change predicted to make a synonymous amino acid substitution, altered splicing by affecting the relative strengths of the spliced forms of the *HGF* gene. The conditional deletion of exon 5 of the *HGF* gene in the cochlea, and apparently no other phenotype, resulted in a profound, nonprogressive hearing loss associated with extensive morphometric pathology [54]. Conversely, the ubiquitous overexpression of HGF in a transgenic mouse resulted in progressive hearing loss associated with a degeneration of the outer hair cells; therefore, the dysregulation of HGF might be involved in nonsyndromic hearing loss.

## 4. HGF as a Biological Drug Candidate

Collectively, tissue-specific disruption of the functional Met in mice indicated that HGF plays a promoting role in the regeneration, protection, and homeostasis of tissues, and an inhibitory role in the progression of chronic inflammation and fibrosis. Thus, enhancement of Met-mediated signaling and biological responses is likely to become therapeutic for the treatment of different types of diseases. In parallel studies addressing the roles of the HGF–Met pathway in genetically modified mice, therapeutic approaches have been pursued in different disease models (Table 2) [55].

The prevention of cell death against various types of stress and injury explains the protective action of HGF, and this seems to be associated with a subsequent suppression of inflammation. HGF regulates the function of dendritic cells and a subset of regulatory T cells [56,57]. The biological actions of HGF on immune cells are likely to underlie the mechanisms by which HGF exerts its therapeutic effect on diseases associated with allergies, inflammation, and fibrosis, at least in part. The promotion of cell proliferation, migration, and 3-D morphogenesis (e.g., branching tubulogenesis in epithelial cells and endothelial cells) driven by the HGF–Met pathway seems to explain the re-organization of tissues from injuries. Chronic tissue injury and inflammation has been associated with the onset of fibrosis. It should be emphasized that there has been no effective medicine for the treatment of chronic fibrotic diseases, whereas HGF-treatment has been effective in reducing fibrosis and improving tissue function in disease models, including liver cirrhosis, chronic kidney disease, dilated cardiomyopathy, lung fibrosis, and vocal fold scarring.

**Table 2:** Therapeutic approaches with recombinant HGF protein in various disease models.

Tissues and Disease/Injury Models	References
**Liver**	
Acute hepatitis	[58,59,60,61,62,63]
Chorestasis	[64]
Fulminant hepatitis	[65,66]
Liver fibrosis/cirrhosis	[67,68,69,70,71]
Liver cirrhosis + surgery	[72]
Alcoholic steatohepatitis	[73]
**Gastrointestinal**	
Ulcerative colitis	[74,75]
Gastric ulcer	[76]
Gastric injury	[77]
**Kidney**	
Acute kidney injury	[78,79,80,81,82,83]
Acute renal inflammation	[84]
Septic acute renal failure	[85]
Diabetic nephropathy	[86]
Chronic kidney disease	[87,88,89,90]
Glomerulonephritis	[91]
Chronic allograft nephropathy	[92]
**Cardiovascular**	
Critical limb ischemia	[93,94,95]
Neointimal hyperplasia	[96]
Coronary artery disease	[97]
Myocardial infarction	[98,99]
Cardiac allograft vasculopathy	[100]
Dilated cardiomyopathy	[101]
**Respiratory**	
Acute lung injury	[102]
Ischemia-reperfusion	[103]
Lung fibrosis	[104,105,106]
Pulmonary emphysema	[107]
Left peumonectomy	[108]
Allergic airway inflammation	[109]
Vocal fold scarring	[110,111]
**Skin**	
Wounding	[112,113]
**Nervous System(s)**		
Cerebral ischemia	[114,115,116,117,118]
Peripheral nerve injury	[119]
Amyotrophic lateral sclerosis	[120]
Hydrocephalus	[121]
Retinal injury	[122]
Photoreceotr degeneration	[123,124]
Difficulty in hearing	[125]
**Musclosleletal**	
Articular cartilage injury	[126]
Skeletal muscle injury	[127]
Rheumatoid arthritis	[128]
Ligament injury	[129]

The administration of HGF exerts neuroprotective effects in the animal models of cerebrovascular diseases, spinal cord injury, and in neurodegenerative diseases including amyotrophic lateral sclerosis (ALS) and neuroimmune diseases by promoting neuronal cell survival and the functioning of glial, vascular, and immune cells. Transgenic expression of HGF in neurons has exhibited beneficial roles in the animal models of Alzheimer’s disease and polyglutamine diseases [43,130]. Experimental allergic encephalomyelitis that is induced either by immunization with myelin oligodendrocyte glycoprotein peptide or by the adoptive transfer of T cells, which mimics multiple sclerosis, was inhibited in a selective overexpression of HGF by neurons in the central nervous system in mice [57]. HGF has mediated mesenchymal stem cell-induced recovery in multiple sclerosis models [131]. Furthermore, systemic HGF treatment has ameliorated experimental allergic encephalomyelitis through the development of tolerogenic dendritic cells [132]. These findings have demonstrated the roles of HGF in the animal models of multiple sclerosis via functioning on not only neural cells but also mesenchymal stem cells and immune cells.

## 5. Clinical Study and Drug Development

### 5.1. Chronic Leg Ulcer

Chronic leg ulcer treatment in elderly patients is a significant healthcare problem. These leg wounds result from different causes, such as diabetes and/or inappropriate circulation, and can be very difficult to heal. Between 1.5 and 3.0/1000 people have active leg ulcers. At present, there has been no satisfactory treatment for many of these patients. The methods available are often time-consuming, difficult, and costly. Involvement of the HGF–Met pathway in the pathophysiological roles of HGF might be considered by evaluating the Met activation status in tissues with pathology. In clinical studies in patients with skin ulcer, the phosphorylation of Met was most prominent in keratinocytes and dermal cells in normally healing wounds; however, tyrosine phosphorylation of Met has been barely detectable in non-healing wounds, suggesting reduced Met activation [133].

The disruption of functional Met in epidermal keratinocytes has indicated that Met-deficient keratinocytes were unable to contribute to the re-epithelialization of skin wounds in mice models [28]. Therefore, activation of the HGF–Met pathway is essential for a fundamental regenerative process during skin wound healing and may not be substituted by other bioactive molecules for the generation of hyperproliferative epithelium in skin. In a full-thickness cutaneous excision model in diabetic mice, topical administration of recombinant HGF protein promoted angiogenesis, extracellular matrix remodeling, re-epithelialization, and wound closure [112,113].

The first clinical study using recombinant human HGF protein was done to investigate the physiological and therapeutic effects of HGF on chronic leg ulcers. HGF in gel form was locally applied to chronic leg ulcers in 11 patients [134]. This pilot study observed that excellent (84%–100% area reduction) or partial healing (58%–59%) was seen in eight out of 11 patients. Significant microcirculatory perfusion was statistically correlated to ulcer area reduction in the treated ulcers. This study suggested that topical application of HGF protein might have facilitated the healing of chronic leg ulcers, possibly by improving the microcirculation. Because no control group was included in this pilot study, proper control studies must be performed for further clinical evaluation.

### 5.2. Critical Limb Ischemia

Critical limb ischemia, the most severe form of peripheral arterial disease due to atherosclerosis, is a common clinical problem that has no effective medical therapy. The standard therapy for critical limb ischemia remains to be lower-extremity revascularization, either through open bypass surgery or by endovascular techniques, or lower-extremity amputation when revascularization is not an option. At present, there is a need for less invasive therapies to improve limb perfusion in patients with critical limb ischemia.

**Figure 4:**
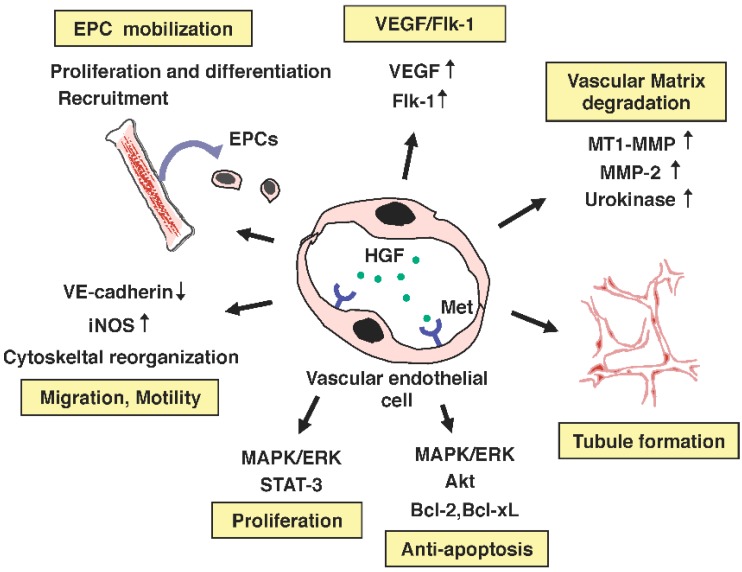
Biological responses leading to angiogenesis driven by the HGF–Met pathway.

Met receptor is expressed in different types of vascular endothelial cells, and HGF stimulates the migration and motility of different types of endothelial cells [135,136,137]. Further studies have indicated that HGF supports angiogenesis through multiple mechanisms, targeting not only endothelial cells but also endothelial progenitor cells (Figure 4). In preclinical animal models for limb ischemia, intramuscular administration of either recombinant HGF protein or expression plasmid for HGF has facilitated collateral new vessel formation, improved blood flow, and reduced muscle atrophy [93,94]. Thus, HGF is a powerful angiogenic growth factor that is applicable for therapeutic purposes.

The first clinical study of *HGF* gene therapy by naked expression plasmid was done to investigate its safety for the treatment of patients with arteriosclerosis obliterans, or Berger’s disease [138]. Subsequently, a multicenter, randomized, double-blind, and placebo-controlled clinical trial was performed for the treatment of patients with critical limb ischemia to evaluate the efficacy and safety of *HGF* gene therapy using naked plasmid [139,140]. This *HGF* gene therapy was proven safe and effective for critical limb ischemia. Phase-II and phase-III clinical trials of *HGF* gene therapy for the treatment of peripheral arterial disease have been completed in the USA and in Japan, respectively.

### 5.3. Hepatitis and Acute Kidney Injury

Conditional Met disruption in hepatocytes indicated they were highly susceptible to apoptosis even in mild injury, and the regenerative response in the liver was retarded when mice were subjected to liver injury. In preclinical models for fulminant hepatitis, systemic administration of recombinant HGF suppressed the onset of fulminant hepatitis [141]. In a similar manner, conditional Met disruption in mature renal cells indicated mice had no appreciable defect in renal function, whereas they were susceptible to severe renal dysfunction and pathology when subjected to renal injury. In preclinical animal models for acute kidney injury, systemic administration of recombinant HGF suppressed tubular cell apoptosis and renal dysfunction, and promoted regenerative cell proliferation [141]. HGF protein may be applicable to the treatment of patients with acute hepatitis or acute kidney injury.

The phase-I clinical trial of the systemic administration of recombinant HGF protein has been completed in both Japan and the USA. In phase I/II clinical trials, intravenous administration of HGF protein was well tolerated by patients with fulminant hepatitis [142].

### 5.4. Amyotrophic Lateral Sclerosis (ALS)

ALS is a fatal neurodegenerative disease characterized by progressive loss of motor neurons and degeneration of motor axons. Approximately 5%–10% of patients have familial ALS, and of those ~15%–25% carry a mutation(s) in the gene encoding Cu^2+^/Zn^2+^ superoxide dismutase (SOD1). Neurons with transgenic expression of mutant SOD1 develop the typical deficits found in both familial and sporadic ALS. Because motoneuronal degeneration is thought to be a primary event in disease progression, treatment approaches have focused on promoting the survival, or at least preventing the death, of motor neurons. In addition to motor neurons, since a reduction in the astrocyte-specific glutamate transporter has been found in ALS patients, astrocytes also seem to be potential targets for ALS therapy.

As described, HGF exerts potent neurotrophic action to promote the survival of a variety of neurons, including motor neurons. The first implication that HGF might be a therapeutic agent for the treatment of patients with ALS was obtained by transgenic over-expression of HGF in the nervous system in mouse model for ALS with mutant SOD1 [143,144]. Neuronal overexpression of HGF has attenuated motor neuron death and axonal degeneration and prolonged the life span of ALS model mice. HGF expression retained the levels of the astrocyte-specific glutamate transporter in reactive astrocytes. Thus, HGF seems to alleviate the symptoms of ALS by direct neurotrophic activities on motor neurons and indirect activities on astrocytes, by reducing glutamatergic neurotoxicity.

Preclinical study to address the therapeutic action of HGF was done using rat model of ALS with mutant SOD1 [120]. In that study, recombinant HGF protein was administered locally into the medullary cavity. The continuous intrathecal administration of HGF attenuated motor neuron degeneration and prolonged the duration of the disease by 63%, even after administration from the onset of paralysis. The phase-I clinical trial for the intrathecal administration of recombinant HGF protein for the treatment of patients with ALS is ongoing at Tohoku University in Japan.

### 5.5. Spinal Cord Injury

Spinal cord injury (SCI) is followed by secondary degeneration, which is characterized by progressive tissue necrosis. Many therapeutic interventions using neurotrophic factors or pharmacological agents have focused on secondary degeneration after SCI to reduce damaged areas and promote axonal regeneration and functional recovery. The therapeutic action of recombinant HGF protein was tested in a primate (common marmoset) model of contusive cervical SCI [145]. Intrathecal HGF administration preserved the intact spinal cord parenchyma, corticospinal fibers, and myelinated areas, thereby promoting functional recovery. HGF-treatment did not give rise to an abnormal outgrowth of calcitonin gene-related peptide positive fibers compared with that seen in the control group, indicating that this treatment neither induced nor exacerbated allodynia. The phase-I/II clinical trial for the intrathecal administration of recombinant human HGF protein for the treatment of patients with SCI was initiated at Keio University in Japan.

## 6. Small-Molecule HGF-Inducers and Therapeutic Approaches

The gene expression of HGF is regulated by growth factors, cytokines, and prostaglandins. Among these, prostaglandin receptors are G-protein-coupled receptors from which different effectors and signaling pathways are evoked. EP_2_ and EP_4_ receptors for prostaglandin E_1_/E_2_ (PGE_1/2_) and prostacyclin receptor (also known as PGI_2_ receptor or IP receptor) for PGI_2_ activate adenylate cyclase upon ligand binding, thereby increasing intracellular cAMP levels. Prostaglandins and their analogs that activate EP_2_, EP_4_, and IP receptors induce transcriptional activation of HGF [146]. On the other hand, transgenic mice that overexpressed COX-2 in hepatocytes were resistant to liver injury [147]. Mice with disrupted cyclooxygenase-2 (*COX-2*) genes, which is a key gene for the production of prostaglandins, developed much more severe liver damage than wild-type mice in a hepatic injury model [148]. The results of these studies have implicated prostaglandins and prostaglandin analogs as being capable of activating EP_2_, EP_4_, or IP receptors, which may exert therapeutic action for pathology and injuries by enhancing endogenous HGF expression.

ONO-1301 was developed as a new type of PGI_2_/IP receptor agonist lacking the typical prostanoid structures [149]. PGI_2_ and its analogs are not stable *in vivo*, whereas ONO-1301 is chemically and biologically more stable than prostacyclin and its analogs because of the absence of prostanoid structures. The therapeutic actions of ONO-1301 and the involvement of HGF have been demonstrated in injury and pathology models in different tissues. In mice models of hepatotoxin-induced liver injury, the administration of ONO-1301 increased the hepatic expression of HGF and suppressed the hepatocyte death and the onset of liver injury [150]. However, the actions of ONO-1301 to suppress hepatocyte apoptosis and to expand necrotic areas were cancelled via the neutralization of endogenous HGF. These results indicate that ONO-1301 increases the expression of HGF and that the hepato-protective action of ONO-1301 against liver injury may be largely attributable to HGF.

ONO-1301 enhanced angiogenesis in the subcutaneous sponge discs of mice, whereas it was mostly inhibited via the neutralization of HGF [151]. In an obstructive nephropathy model, a single injection of sustained-release ONO-1301 suppressed fibrogenic gene expression and increased renal HGF expression, and this was associated with a suppression of interstitial fibrosis of the kidney [152]. These therapeutic effects of ONO-1301 were significantly cancelled via the neutralization of HGF. In a rat model of myosin-induced experimental autoimmune myocarditis (the heart transits from an acute inflammatory phase to a chronic dilated cardiomyopathy phase in this model), the oral administration of ONO-1301 increased capillary density in the myocardium and circulating endothelial progenitor cells, and improved hemodynamic functions, and these beneficial effects of ONO-1301 were partially abrogated via the neutralization of HGF [153]. In an experimental chronic asthma model, the subcutaneous administration of ONO-1301 increased pulmonary HGF expression, suppressed airway hyperresponsiveness, and improved airway remodeling/fibrotic change, while neutralization of HGF significantly abrogated the effects of ONO-1301 [154]. In a model of pulmonary arterial hypertension, the oral administration of ONO-1301 significantly attenuated the increases in right ventricular systolic pressure and the increases in the medial wall thickness of pulmonary arterioles [155]. The neutralization of HGF together with ONO-1301 cancelled the improvement in survival rates that had been achieved by ONO-1301 alone, which suggested the involvement of HGF in the therapeutic action of ONO-1301.

These studies using ONO-1301 PGI_2_/IP receptor agonist demonstrated the therapeutic approach of using a small molecule capable of inducing HGF, as a regenerative medicine. The use of such HGF-inducible small molecules seems to have an advantage in terms of versatility as a medical drug, including oral administration and a combination of drug delivery techniques.

## 7. Conclusions

Based on the close involvement of HGF and its receptor Met—not only in tumor development, invasion, and metastasis but also in resistance to anticancer therapies—drug discovery targeting the HGF–Met pathway has become a hot target in anticancer drug development [156,157,158,159,160]. On the other hand, based on studies using cell/tissue-specific disruption of functional *Met* and preclinical disease models in experimental animals, recombinant HGF proteins and *HGF* genes have become biological drug candidates for the treatment of patients with diseases marked by impaired tissue function [43,141]. Thus, both selective and appropriate inhibition and activation of the HGF–Met pathway are therapeutic approaches that are based on a scientific basis.

Growth factors and cytokines have profound biological and physiological activities, and several growth factors and cytokines have been used as medical drugs: insulin-like growth factor-1, granulocyte-colony stimulating factor, erythropoietin, interferon-γ, and fibroblast growth factor-2. These cytokines and growth factors exert a characteristic pharmaceutical efficacy, and therefore cannot be replaced by other small molecules and chemical compounds.

The characterization of mice with cell/tissue-selective disruption of the *Met* gene particularly revealed the irreplaceable role of the HGF–Met pathway in survival, regeneration, and homeostasis of cells/tissues, which thereby provides a rationale for the therapeutic use of HGF. An appropriate activation of the HGF–Met pathway is performed by different approaches, such as recombinant HGF proteins, *HGF* genes (expression vector for *HGF* genes), and small-molecule HGF inducers. These approaches have different advantages and characteristics for medical use. Because clinical development of the *HGF* gene and recombinant HGF protein as a regeneration-based drug candidate has progressed and is ongoing, proof-of-concept is expected to be established after further clinical trials.
